# An analysis of the relationships between human, technological and physical factors in the retail banking sector

**DOI:** 10.1007/s43039-022-00048-x

**Published:** 2022-01-31

**Authors:** Michela C. Mason, Francesco Massara, Francesco Raggiotto

**Affiliations:** 1 University of Udine, Via Tomadini 30/A, 33100 Udine, UD Italy; 2 Università IULM, Via Carlo Bo, 1, 20143 Milano, Italy

**Keywords:** Self-service technologies, Customer behaviour, Customer satisfaction, Banking services

## Abstract

In the light of the increasingly massive implementation of technology in retail settings, the present research aims at exploring the relationships between interacting factors of the retail servicescape: human interaction, automated service, and atmosphere, and their impact on customer satisfaction regarding the service. We develop a theoretical framework to understand the relationships between the single components of the servicescape and we empirically test our framework within the context of retail banking services. We develop a moderated mediation model on a sample of 1346 retail banking customers. We find that the human factor mediates the relationship between self-service technologies and satisfaction, and that this mediation is negatively moderated by a favourable perception of the banking service atmosphere. Theoretical and managerial implications are discussed.

## Introduction

Technological integration is, by far, one of the major challenges service providers are facing nowadays. Technology, in various forms (e.g., service automation, digitalization, and so forth), is often at the core of the redesign of value propositions in the service sector, in terms, for instance, of efficiency (e.g., Meuter et al., 2005), quality (e.g., Weijters et al., 2007), and of multichannel customer experiences (e.g., Baier & Rese, 2020; Hu & Tracogna, 2021).

Self-Service Technologies (from now on, SSTs) are the most ubiquitous technologies in the service landscapes (Sharma et al., 2021). Their popularity largely resides in the advantages they offer both for customers and for companies. For instance, the former can save time, obtain improved service standards, and increase control over the service production process; the latter reduce costs (particularly, labor costs), improve service quality as well as achieve higher levels of customer satisfaction and loyalty (e.g., Demirci Orel & Kara, 2014; McWilliams et al., 2016).

With technological advancement moving at an increasingly fast pace and technology being increasingly accessible to customers and providers, the major challenge becomes, indeed, not the one of technology appropriation and implementation, rather the one of technology integration of technology within service environments (Inman & Nikolova, 2017). In other words, it is the way in which technology integrates in service environments which qualifies its ultimate contribution in terms of competitive advantage. In this sense, SSTs represent a case in point: technological advancement allows SSTs to cover a wide array of applications, from the simple product scan and checkout procedures (e.g., Rinta-Kahila et al., 2021), to the full replacement of the human presence within the retail setting, e.g., by means of interactive machines or even robotic interfaces (Shin & Perdue, 2019).

In the past 2 decades, the debate on SSTs in marketing and consumer research revolved around some well-identified areas, which mostly explored the way in which consumers relate to SSTs: the antecedents of consumer adoption of SSTs (e.g., Collier et al., 2015; Oghazi et al., 2012; Wang et al., 2012), and their satisfaction related to technology usage (e.g., Narteh, 2015). While extant research on the factors which determine consumers reactions regarding SSTs appears well developed, more recently, research has called for developing a broader understanding of the ways in which technological adoption is reshaping the overall relationship between service providers and customers, in terms, for instance, of overall customer evaluations and responses (e.g., Dekimpe et al., 2020; Sharma et al., 2021).

In terms of SSTs research, of particular importance is exploring the relationship between SSTs and the human factor. Scholars consistently reported that the success (and failure) of SSTs introduction within a service environment may frequently depend upon how SSTs relate with the human presence within the environment (e.g., Bulmer et al., 2018; Sharma et al., 2021). For instance, there is evidence of the fact that failure of technology introduction in service settings may be mostly due to the lack of social interaction with other people in the service encounter (Scherer et al., 2015); indeed, the presence of technology, without any kind of “human touch” within the service environment may simply be not be enough to achieve the goal of cross-selling or up-selling, which, rather, much more rely upon the action of individuals (e.g., Lee & Coughlin, 2015). Accordingly, it has been suggested that, simply considering technology as a mean of replacement of other service components (most notably, humans) may reveal as a myopic strategy, expose companies to potential competitive failure (Mukerjee, 2020).

In this research, we take on this suggestion to investigate the effects of the technological integration within service settings in terms of the overall relationship between the customer and the service provider. We observe the effects of the customer’s simultaneous interactions with technology and employees on the overall service experience; furthermore, extending recent suggestions in the research (e.g., Choo & Petrick, 2014; Kaminakis et al., 2019), we explore the effects of servicescape on the above-mentioned interactions.

We suggest that for consumers to accept the substitution of employees with a computer-mediated interface, they need to understand that such a substitution will improve their relationship with the service provider: in particular, that technology is there to empower, not just eliminate, the human component of that service. Given that relationships with frontline employees (and evaluation thereof) make a key contribution to service performance, contributing, for instance, to intensify value for consumers (Morhart et al., 2009; Sirianni et al., 2013), and, ultimately, to reinforce relationships with customers (Solnet et al., 2019). Our research is set in retail banking services, a kind of high-contact, utilitarian service setting. Retail banking is among the service industries which invest in SSTs since the early 1970s (NCR, 2021). While early applications of SSTs mostly focused on improving cost effectiveness and customer convenience of service delivery (while at the same time allowing companies to exploit relevant economies of scale, (Wirtz & Zeithaml, 2018), more recent applications emphasized more the augmenting role of SSTs of those services traditionally delivered by customers (Dauda & Lee, 2015), redesigning customer service managed by employees as a differentiating factor (Shin et al., 2017).

The remainder of the paper is organized as follows. First, we present the theoretical foundation of our conceptual framework and the rationale underlying the relationships between the proposed constructs. Then, we present the methodology of our study, the analyses, and the results. Finally, we provide some implications for research and for banking practitioners.

## Theoretical background

In technology-enabled service encounters, the service process has two components: the technology service process and the human service process. Both help shape consumer perceptions (Makarem et al., 2009). The technology service process typically employs SST, defined as a “technology interface that enables customers to access a service independent of direct service employee involvement” (Meuter et al., 2000, p. 50).

Traditionally, the literature considered service encounters as essentially shaped by human interactions (Bitner, 1992; Czepiel, 1990). However, the recent massive infusion of technology into service encounters has led researchers to consider that consumers’ service experiences are shaped not only by human interactions but also by the interactions between consumers and service technologies (e.g., interactions with SSTs; Sharma et al., 2021; Svensson, 2006). Even in the face of relevant service technology integration, the human touch still appears crucial in delivering service experiences. For instance, Frontline Employees (FLEs thereafter) are a source of unique value—for example, the relational and emotional value—for customers that automated technology is often unable to deliver (Solnet et al., 2019). FLEs can adapt to changing customer needs (Rego et al., 2014), crafting a unique service; they can detect latent needs of customers, and develop strong bonds with them (Coelho et al., 2011); and, through them, customers assimilate brand personality (Sirianni et al., 2013). In other words, FLEs help augment service standards (Ottenbacher & Harrington, 2009), deliver superior customer experiences, and strengthen long-term customer relationships (Coelho et al., 2011).

Researchers have recently begun to investigate the limitations of service technology integration that ignores the centrality of human interactions. For example, scholars have warned that a massive implementation of technology in service settings may lower perceived customer service, depersonalizing the service atmosphere (Alpert, 2008).

Because of the dyadic nature of service encounters (Solomon et al., 1985), FLEs are key in shaping service experiences and importantly contribute to shaping customers’ perceptions of service quality (Alexiadou et al., 2017; Svensson, 2006).

We thus propose a revised servicescapes framework in which, with respect to the original (Bitner, 1992), we include SSTs that become a factor interacting with both employees and customers. SSTs are inserted in a service environment within an atmosphere that envelops the triangulation between FLEs, SSTs, and customers.

## Hypotheses development

Satisfaction reflects a positive sentiment regarding a service encounter, derived from the assessment of the extent to which service performance meets expectations (Zeithaml et al., 2003). There is consistent evidence that consumer usage of SSTs can affect customer satisfaction (e.g., Demirci Orel & Kara, 2014; Taillon & Huhmann, 2019). For instance, in a study on self-checkout, Fernandes and Pedroso (2017) found that perceptions of SSTs predict overall customer satisfaction, a finding that has been confirmed across diverse sectors (e.g., Bogicevic et al., 2017) and even in online service settings Boon-itt, 2015). In a similar vein, Beatson et al. (2007) considered that the performance of SST attributes will have an effect on overall satisfaction, although this effect may be moderated by the frequency of SSTs usage. Djelassi et al. (2018) investigated how the evaluation of SST experience affects customer satisfaction, finding that satisfaction with SSTs strongly mediates the effect of SST experience evaluation on overall store satisfaction. In a study of service failure and recovery in using SSTs, Dabholkar and Spaid (2012) found that immediate recovery of SST failures increased customer/user satisfaction with the experience and that SST errors (as opposed to user errors) decreased user satisfaction.

It is reasonable to expect that in the banking sector as well consumer overall satisfaction is impacted by technology usage. In the last decades, the banking service sector has extensively embraced technological innovation, widely introducing technology into processes, procedures, and in developing relationships with customers (e.g., Adapa & Roy, 2017; Kaur & Ali, 2021); in this sense, SSTs play a key role in the banking service sector, as in recent years, the banking industry has widely implemented self-service technologies (SSTs) in service encounters (FinTech Futures, 2018; Mukerjee, 2020).

Basing on the above discussion, we therefore advance the following hypothesis:

### H1

Consumer perceptions of SSTs have a direct effect on consumer overall satisfaction.

The relationships established between customers and service employees are central in the development of service satisfaction (e.g., Gwinner et al., 1998; Kwortnik, 2008).

Studies in the banking sector noted that human rapport is crucial regardless of the level of technology implementation: consumers perceive human interaction as key to accomplishing goals in banking service encounters, which they generally perceive as complex. Further, human interaction, as a dimension of the overall service experience, is more relevant than technological factors (Bell & Eisingerich, 2007) and may contribute to consumers’ negative as well as positive attributions. Dabholkar and Spaid (2012) found, for example, that FLEs’ active intervention to resolve SSTs failure increased negative attributions to the SST. Collier et al. (2017), on the other hand, found that customers want employees to fully take over a transaction after a failure, and that if employees do so, customers are less likely to switch to a full-service option on their next visit to the retailer. Overall, there is wide evidence that in banking service settings the human service component contributes crucially to the establishment of trust between the bank and their customers, a key determinant of consumer satisfaction and future intentions (Shainesh, 2012). Arguably, prior development of trust is a fundamental condition for banking customers to approach technology-based banking services (e.g., e-banking services). Therefore, even if a specific episode of service recovery led by FLEs has a negative effect on SST (Dabholkar & Spaid, 2012), here we consider, more in agreement with Collier et al. (2017), that a positive overall contribution of the human factor to the quality of the environment—and thus not related to a single episode—has a positive halo effect on the usage and satisfaction with SST. More specifically, the presence of an employee mediating/supporting may reinforce positive consumer perceptions related to SSTs (e.g., regarding privacy or security perceptions; cf. (Shainesh, 2012), complement consumers’ perceived technological self-efficacy (Immonen et al., 2018), and/or counter the frustration/dissatisfaction generated by negative consumer–service technology interactions (Larivière et al., 2017). We therefore expect that in banking services, the perception of the overall quality of service by FLEs may contribute to consumers’ overall perceptions related to SSTs. Hence, we propose the following:

### H2

Consumer perceptions of FLEs mediates the relationship between consumer perceptions of and overall satisfaction.

Service atmosphere is a determinant of servicescapes, along with the human factor and self-service technologies. Its effect is subtle and indirect, as it interacts with all other elements of the service environment contributing to the customer’s emotional experience (Massara & Pelloso, 2006). The marketing and retailing literature emphasizes the role of the atmosphere or store environment as the “pleaser and teaser” of shopping (Elmashhara & Soares, 2022; Kotler, 1973; Roggeveen et al., 2020; Turley & Milliman, 2000). The store environment is a container of “must haves” but also a potential carrier of “wow effects” and constitutes a filter for the overall emotional experience. According to Bitner (1992), servicescapes facilitate positive encounters and interactions between frontline personnel and customers, ultimately supporting the path-to-purchase. Positive reactions to the atmosphere create the basis for the consumer’s approach behaviour and positive response (Donovan et al., 1994). In the context of this paper, we are interested in highlighting evidence of the influence of atmospherics, let alone the human factor, on perceptions that may facilitate adoption of SSTs, such as development of trust, safety, control, and security, which in the banking context may justify the preference for or the mediation of human FLEs. Such evidence exists; Dabholkar and Spaid (2012) found that a low-anxiety atmosphere—described to customers as a “quiet, out-of-the-way area, with a phone handy to call for assistance if needed” (p. 1421) and thus where the customer felt like they were in control—is associated with lower negative attributions to the SST, as well to any employee who tried to assist, and to the retailer. Moon et al. (2017), in the context of airports, a context in which safety is relevant to the customer experience, as in banks, found that environment aesthetics has many positive externalities for perceptions of safety and cleanliness and a very strong impact on satisfaction and behavioural intentions.

In recent years, banks have devoted increasing effort to redefine their value proposition. Since the early 1990s (e.g., Greenland, 1994) banks have put great efforts in constantly modernizing their physical presence, which, regardless of technological evolution, still remains a crucial component of the overall customer experience (Allard et al., 2009; Forbes, 2021; Reydet & Carsana, 2017). In banking settings, atmospherics have been identified as a key tool for creating customer affection, loyalty (e.g., Reydet & Carsana, 2017) and contributing to the overall customer satisfaction (Iglesias et al., 2019). There is consistent evidence of the fact that environmental components can affect customers even before the actual service performance, e.g., determining consumers’ pre-consumption mood (Mattila & Wirtz, 2001; Namasivayam & Mattila, 2007). Regarding banking settings, Greenland and McGoldrick (2005) suggested that, in banking settings, a key role of atmospherics concerns enhancing (or reducing) customers’ perception of safety within the bank branch, which can be therefore perceived as “more approachable, less dominant and less crowded” (Greenland & McGoldrick, 2005, p. 146). This evidence suggests that atmospherics may help develop a sense of security in consumers, which may reverberate on all the in-branch consumer interactions, including SSTs usage. Regarding SSTs usage, atmospherics may soothe consumers’ resistance towards technology, which in the context of self-service banking may well have to do with issues of safety and security, therefore reducing the need for a reassuring human intervention.

Basing on the above discussion, we propose the following:

### H3

Service atmospherics moderates the mediation between consumers’ SST perception and FLEs perceptions.

## Methodology

### Setting and sample

In the banking sector, service encounters are characterized by several applications of SST, which either complement banking services (e.g., ATMs) or fully replace employee interactions (Blut et al., 2016). We interviewed 1,346 customers of a large European bank using a paper-and-pencil questionnaire. The subsidiaries selected in our field study make use of advanced ATMs, which allow bank customers to access diverse banking services, like: check deposit, checking account movements, access mobile top-up services and bank transfer services. Interaction with bank employees complements the presence of machines through value-added services like consultancy and commercial services.

Bank respondents were asked to respond to the questionnaire while thinking about their banking experiences and to assess their satisfaction with the service provided by the bank, its employees, the available SSTs, and the banking service atmospherics. The questionnaire asked respondents about service personnel (10 items from Ladhari et al., 2017; we adapted the sentences to the banking setting), in-store atmospherics (3 items from Ladhari et al., 2017), and perceptions related to the usefulness of SSTs (10 items from Inman & Nikolova, 2017; we adapted the sentences to the banking setting). Then, respondents were asked how satisfied they were with the service experience (4 items from Picón et al., 2014; we adapted the sentences to the banking setting). All survey items were measured using 7-point Likert scales.

### Measurement instrumentation and model estimation

Measurement adequacy was checked by estimating convergent validity, through item reliability, construct reliability (CR) and average variance extracted (AVE). Regarding item reliability, a factor analysis using maximum likelihood and varimax rotation with SPSS 25 showed that the items loaded onto four factors, explaining over 70% of the variance (Hair & Lukas, 2014). All factor loadings exceeded the recommended 0.6. threshold (Bagozzi & Yi, 1988), thus supporting convergent validity (Table 1). Support for construct reliability, was found in CR values and Cronbach’s alphas values, that were all higher than the recommended threshold of 0.7 (Table 2).

**Table 1:** Questionnaire items

	Loadings
*FLE perceptions*	
1. The bank employees get things right the first time	.891
2. The bank employees provide prompt services	.915
3. The bank employees are always willing to help me	.909
4. You can trust the bank staff	.922
5. You feel safe/confident dealing with the bank staff	.898
6. The bank staff are courteous	.913
7. The bank staff have the knowledge to answer your questions	.885
8. When I have a problem, bank staff members show an interest in resolving the problem	.869
9. The bank provides its services on time	.891
10. The staff members at the bank know your specific needs	.891
*Overall satisfaction*	
1. I am satisfied with my decision to go to this bank	.934
2. If I had to do it all over again, I would go to this bank	.946
3. My choice to go to this bank was a wise one	.945
4. I think that I did the right thing when I decided to go to this bank	.925
*Service atmospherics*	
1. Irritating atmosphere/soothing atmosphere	.931
2. Bad scent/Good scent	.908
3. Unpleasant light/pleasant light	.921
*SST perceptions*	
1. Given the investments I need to make to adopt this new technology (e.g., time, personal information, money), the final outcome that I will receive is fair	.921
2. The outcome of the bank’s implementation of this new technology is very positive for me	.813
3. Considering the inconvenience that this technology might cause me, the outcome that I will receive is more than fair	.850
4. My commitment to continue my relationship with the bank	.918
5. My belief that my relationship with this bank deserves my maximum effort to maintain	.927
6. My intent to maintain my relationship with this bank indefinitely	.892
7. My loyalty towards this bank	.938
8. The extent to which I care about the long-term success of this bank	.916
9. My overall satisfaction with the bank	.902
10. My intent to maintain my relationship with this bank indefinitely	.832

RMSEA = 0.078; p(RMSEA < 0.05) < 0.001; CFI = 0.93; NFI = 0.91

**Table 2:** Measurement properties

	Cronbach’s alpha	Rho	CR	AVE
Overall satisfaction	0.954	0.954	0.967	0.879
FLE perceptions	0.973	0.974	0.977	0.807
Service atmospherics	0.910	0.916	0.943	0.847
SST perceptions	0.971	0.975	0.975	0.796

Regarding discriminant validity, it exists if the minimum AVE exceeds the squared correlation between two variables. In this case, the minimum AVE is 0.80, while the highest squared correlation between any two variables is 0.50 (Table 3). Discriminant validity of the constructs is thus ensured. After checking for convergent validity and discriminant validity, we tested the relationships between the variables.

**Table 3:** Correlations

	Satisfaction	FLE perceptions	Service atmospherics	SST perceptions
Overall satisfaction	1			
FLE perceptions	0.709	1		
Service atmospherics	0.597	0.482	1	
SST perceptions	0.340	0.318	0.243	1

The theoretical model was estimated by means of a structural model using partial least squares structural equation modelling (PLS-SEM). PLS-SEM is a second-generation, variance-based estimation procedure which uses a set of ordinary least squares analyses (Kiani & Laroche, 2019). PLS-SEM does not consider any assumption regarding joint distribution of indicators or regarding independence of sample cases (Chin, 1998).

PLS estimating iterative algorithm first solves the measurement model blocks; then, it estimates the path coefficients of the structural model (Iglesias et al., 2019). Literature reports that PLS-SEM is a particularly useful procedure in the case of testing complex models and relationships between constructs (Chin, 1998; Kiani & Laroche, 2019). As suggested by other scholars (e.g., Iglesias et al., 2019), the PLS-SEM method appears appropriate for this research in that the proposed model is complex (involving 27 items for 4 constructs) and contains complex relationships (both mediators and moderators).

The model was tested using the SmartPLS 3.0 program. As a second-generation method of estimation, unlike other programs (e.g., SPSS AMOS), SmartPLS allows the direct inclusion and measurement of moderator effects into the model (Kiani & Laroche, 2019).

**Fig. 1:**
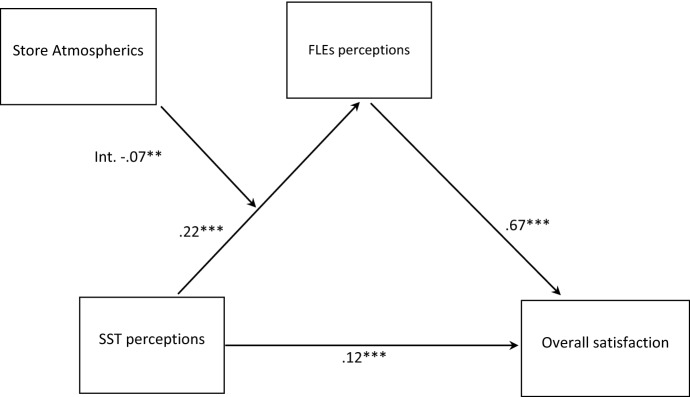
The model with estimates

Hence, using the features included in the software, SSTs perception was entered as the dependent variable; overall customer satisfaction was the dependent variable, while FLEs perception was entered as the mediating variable between SSTs perception and overall customer satisfaction. Then, the hypothesized moderating effect was included in the model (Table 3). Specifically, store atmospherics was entered as a moderator of the relationship between SSTs perception and FLEs perception (see Fig. 1).

## Results

Model estimation showed a significant direct effect for SSTs perception on satisfaction (β = 0.12, *p* < 0.001), thus supporting H1; positive SSTs perception lead to positive FLEs perception (β = 0.22, *p* < 0.001); that FLEs perception positively impacts satisfaction (β = 0.67, *p* < 0.001), thus supporting *H2.* Model estimation hence supports a partial mediation of FLEs perception in the relationship between SSTs perception and consumers’ overall satisfaction.

Furthermore, as we advanced in *H3*, Service atmosphere moderates the mediation between SST perception and FLEs perception (Effect = − 0.07, *p* < 0.05). thus, H3 is statistically significant as well, with negative sign.

In summary, H1–H3 are supported, suggesting that customers’ perceptions of service technology (i.e., SST), coupled with customers’ perceptions of service employees (FLEs), drive ultimate satisfaction with the service. Nonetheless, technology appears pervasive in the service encounter, directly influencing the overall service experience. More positive perceptions related to service atmosphere reduce the importance of human mediation in determining the influence of technology on service satisfaction.

The results of the PLS-SEM estimation are illustrated in Fig. 1.

## Discussion and implications

Both industry practitioners and academics converge in suggesting that, the key challenge for banks is the issue of technology integration rather than the simple substitution of humans with technology. The results of the present study contributes to the scholarly debate on the role of technology in service encounters. Whereas the issue of technology substituting for human service is central to the marketing, retailing, and service literature (Huang & Rust, 2018), how automated technology—and notably SSTs—may impact consumer outcomes is yet to be fully understood (Ostrom et al., 2019; Sharma et al., 2021). Accordingly, in the attempt to delve into the possible key elements relating to technological integration and its related impacts on consumer evaluations, the proposed model encompassed both moderating and mediating pathways, linking the technological service process (delivered by SSTs) with the surrounding service environment. In line with extant research (e.g., Pooya et al., 2020), our results suggest that consumers interaction with technology in service settings (namely, SSTs) is a primary driver of the overall consumer satisfaction. Notably, results of the present research also suggest, that, in some settings, interaction with technology alone may tell only one side of the story when it comes to considering overall customer satisfaction. Indeed, the impact of consumer interaction with technology is also mediated by consumer perceptions regarding the “human touch”. At first, this result may appear counterintuitive, especially if considering the rationale which frequently guides SSTs adoption in banks (i.e., cutting costs related to personnel). However, such result appears in line with recent insights concerning the role of human presence in banking service settings. Albeit banks are strongly pushing towards digitalization and technology adoption, still, a relevant share of the customer base perceive the physical presence as a key component of the banking service. For instance, in a recent study, Iglesias et al. (2019) noted that, in banking settings, employees empathy is so crucial for consumer evaluation to partially offset the impact of other components of the customer experience. Accordingly, “when employees are empathic […] they become the key driver to customer satisfaction. Consequently, and comparatively, when evaluating the brand experience, customers then pay less attention to the positive sensory cues” (p. 351). Furthermore, if, on the one hand, banks production processes are almost entirely carried out by technology and digitalized (McKinsey & Company, 2020), on the other hand, recent insights from the industry (e.g., Forbes, 2021) suggest that, in designing service encounters, banks can hardly get rid of any physical presence: the majority of bank customers, apart from digital services, still feel the need of a physical space in which cultivate their relationship with the bank. In other words, banks physical presence is something that individuals still expect as part of the service. Indeed, it is how the bank mixes the digital with the human which determines the ultimate outcome in terms of satisfaction. From a practical standpoint, while SSTs may help customers retrieving information regarding banking services, or carry out low value-added operations, still, they are not able to boost the relationship, through, for instance, a reassuring action regarding customer doubts related to data returned by the ATM on his/her bank account (e.g., regarding fees customer is not able, at first, to explain). In other words, results of the present study suggest that human labour substitution may not always be recommendable in technology-based service encounters: rather, even in such encounters, the human touch strongly retains its strategic relevance as a key source of competitive advantage because of its ability to add unique value to technology-enabled services, delivering a “unique dimension to technology, regardless of functionality” (Larivière et al., 2017, p. 241), which may result in unique customer experiences by, for instance, developing customers’ emotional connections with services (Solnet et al., 2019) or, in high-complexity services (like banking), uniquely improving key service perceptions like those related to reliability and safety.

Another relevant finding of this study concerns the role of atmospherics. Results show that atmosphere negatively moderates the mediation between SSTs perception and FLEs perception; in other words, a favourable perception of the service atmosphere diminishes the mediating role of employees. On the one hand, this finding corroborates insights of marketing research (e.g., Elmashhara & Soares, 2022; Roggeveen et al., 2020; Turley & Milliman, 2000); and of consumer research set in retail banking (e.g., Bakar et al., 2017; Hossain et al., 2021) establishing the key role of atmosphere in shaping consumer perceptions; on the other hand, it encourages some reflections about the specific role of atmospherics in retail banking. The retail banking sector is undergoing deep transformations, deeply influenced by technological evolution, and pushed even more forward by the COVID-19 pandemic. Practitioners are urged to refocus their value proposition, toward the creation of compelling customer experiences. In this sense, a key, strategic issue in the retail banking industry concerns the role of bank branches in supporting the post-pandemic repositioning of players in the industry. From a practical standpoint, our results may suggest that, in banking settings, a favourable service atmosphere may improve customer physical banking experience, in that, for instance, it may mitigate consumers’ perceived difficulties (e.g., usage complexity) related to technology adoption, thus reducing the need for a reassuring human intervention. However, our results also highlight the existence of an overall interplay between atmospherics and FLEs, therefore suggesting that the role of atmospherics in banking should not be considered only from the point of view of reducing the importance of FLEs in shaping customer experience (and, consequently, the importance of banks’ investments devoted to FLEs). Rather, it raises attention towards the importance to effectively combine the positive effects of digitalization (e.g., improved access to services for customers at lower costs) with the key features of a more traditional “bricks and mortar” approach, which characterized retail baking for many decades.

In other words, to redefine their value proposition in the market, players may be urged to refocus the role of bank branches, which, rather than being seen as a liability, may instead hold an enormous strategic potential. Operating branches in which technology is not dominating the service environment, rather it is fully integrated in it, may allow banks to attract diverse customer segments (e.g., customers more or less accustomed to technology usage); furthermore, by creating a pleasant atmosphere, banks may succeed ensuring those customers perceptions that are key for the overall customer evaluations of the service (e.g., Bakar et al., 2017; Hossain et al., 2021). Notably, a pleasant environment may not just improve the overall customer evaluation, rather it may also work as a relief for customers, on the one hand encouraging them to experiment technology; on the other hand, allowing banks to release more human resources and focus them on more value-added tasks, like personal consultancy.

Overall, our model may provide some suggestions for the development of a strategic arrangement of the multiple banking service components which may optimize the potential of technology, while at the same time help redefining the role of bank branches and of employees, from a low-value added model to a model aimed at reinforcing the banks’ customer relational capital, a strategy which appears nowadays the most attractive for banks redesigning their value propositions, in the light of the recent financial crises and of the Coronavirus pandemic (Johnson & Peterson, 2014; KPMG, 2016; McIntyre, 2020).

## Limitations and further research

This study has some limitations. First, data were collected in Italy only. Despite the COVID-19 pandemic has boosted access to digital banking services, traditionally, in Italy the role of physical bank branches (and of traditional banking models) appears more relevant in other countries. In this sense, to strengthen the generalizability of the results, further research should examine other countries, possibly denoted by different retail banking models. Further generalization of the results would also benefit from extending the study to multiple banking institutions, as in our study the data were collected from customers of a single, specific national bank. Regarding the components of the model, further research may investigate which specific meanings atmospherics may trigger to encourage SSTs customer usage (e.g., privacy or security, Allard et al., 2009).

Further research could focus on comparing different types of customers (e.g., younger vs. older consumers, and those more vs. less familiar with technology) as well as different types of banks (e.g., small local banks vs. larger national and international banking groups). Technology-enabled service encounters evolve rapidly because of technological advancement. Service providers are investing heavily in the implementation of smart technologies in service encounters. These technologies are designed to substitute for human labour, allowing firms to reduce costs. However, they allow not only the substitution of human labour but also a high level of adaptability to customer needs, requests, and behaviours (e.g., habits, past preferences, and past behaviours). Our research focused on a single technology (SSTs); further research efforts might integrate smarter technologies (e.g., mobile applications) as well as virtual service environments (e.g., virtual servicescapes), which are increasingly relevant for banking services (e.g., due to the massive usage of online banking and other technological tools). Another key limitation of the study concerns the fact that the proposed model provided an overall representation of the interplay between diverse components of the banking servicescape in shaping consumer satisfaction. Indeed, the variables considered in this study are likely to relate differently to each other according to the kind of interaction that occurs in the banking service encounter (e.g., technology-mediated interaction without the intervention of employees; human-based interaction without any kind of technological mediation). In this sense, further research may take into account how relationships between the variables examined in this study vary according to different kinds of customer interaction, and how different relationships between these variables affect customer satisfaction for different kinds of bank–customer interaction.

## Appendix

See Tables 1, 2, 3 and

**Table 4:** Findings

	Model 1	Model 2
Independent variable SST perceptions	0.32***	0.22***
Moderating variable FLEs perceptions		− 0.07**

**p* = .05

***p* = .01

****p* = .001

^4^.
